# Low Herbivory among Targeted Reforestation Sites in the Andean Highlands of Southern Ecuador

**DOI:** 10.1371/journal.pone.0151277

**Published:** 2016-03-10

**Authors:** Marc-Oliver Adams, Konrad Fiedler

**Affiliations:** Department of Botany and Biodiversity Research, Division of Tropical Ecology and Animal Biodiversity, University of Vienna, Vienna, Austria; University of Guelph, CANADA

## Abstract

Insect herbivory constitutes an important constraint in the viability and management of targeted reforestation sites. Focusing on young experimental stands at about 2000 m elevation in southern Ecuador, we examined foliar damage over one season as a function of tree species and habitat. Native tree species (Successional hardwood: *Cedrela montana* and *Tabebuia chrysantha*; fast-growing pioneer: *Heliocarpus americanus*) have been planted among prevailing local landcover types (abandoned pasture, secondary shrub vegetation, and a *Pinus patula* plantation) in 2003/4. Plantation trees were compared to conspecifics in the spontaneous undergrowth of adjacent undisturbed rainforest matched for height and foliar volume. Specifically, we tested the hypotheses that *H*. *americanus* as a pioneer species suffers more herbivory compared to the two successional tree species, and that damage is inversely related to habitat complexity. Overall leaf damage caused by folivorous insects (excluding leafcutter ants) was low. Average leaf loss was highest among *T*. *chrysantha* (7.50% ± 0.19 SE of leaf area), followed by *H*. *americanus* (4.67% ± 0.18 SE) and *C*. *montana* (3.18% ± 0.15 SE). Contrary to expectations, leaf area loss was highest among trees in closed-canopy natural rainforest, followed by pine plantation, pasture, and secondary shrub sites. Harvesting activity of leafcutter ants (*Acromyrmex* sp.) was strongly biased towards *T*. *chrysantha* growing in open habitat (mean pasture: 2.5%; shrub: 10.5%) where it could result in considerable damage (> 90.0%). Insect folivory is unlikely to pose a barrier for reforestation in the tropical Andean mountain forest zone at present, but leafcutter ants may become problematic if local temperatures increase in the wake of global warming.

## Introduction

Although the global rate of deforestation appears to be slowing down, increases in the Earth's forest cover are mostly restricted to higher latitudes and offset by net losses still taking place in the tropics [1, 2]. Many governments are in the process of abandoning or revising policies that actively promote the conversion of forest to agricultural land (Cost Rica: [3], Brazil: [4]), but market forces, population growth, and unsustainable management practices continue to foster agricultural expansion [5]. In Latin America, extensive livestock farming constitutes an important driving force in this regard, as many farmers and low-income smallholders rely on cattle as part of their livelihood [6]. In the equatorial Andean highlands, comparatively poor soil conditions and the intrusion of aggressive weeds in the wake of slash-and-burn management (e.g. *Pteridium arachnoideum*; [7]) often render the sustained use of pastures uneconomic, thus forcing the tenants to periodically clear new land for grazing. Competition by the robust successional shrub/fern community [8] and reduced seed deposition [9] severely impede the course of natural forest regeneration on former pasture land.

While the protection of pristine forests remains essential in the conservation of biodiversity [10, 11], secondary and plantation forests play an increasingly important role in maintaining vital ecosystem services (e.g. conservation: [12], soil protection: [13, 14], carbon sequestration: [15]), emphasizing the need for targeted reforestation of degraded landscapes. In the past, tropical forestry relied heavily on a small number of exotic genera (e.g. Eucalyptus and Pinus spp.; [16]), but in recent decades the focus has increasingly shifted towards the use of native timber species [17, 18] for both ecological [12, 19] and economic reasons (e.g. [20, 21, 22]). Studies have shown that restored sites can be functionally equivalent to natural succession in insect species richness, abundance and herbivory (but not species composition; [23, 24]), and thus capable of upholding vital ecological functions (e.g. food supply for higher trophic levels, regulation of plant community though herbivory). Nonetheless, insufficient knowledge regarding the ecology and management of native tree species currently limits their silvicultural use [25].

The successful establishment of a plantation is not only contingent on species-specific requirements regarding climate and soil conditions [16], but also on herbivore pressure at the prospective site with woody plants as a whole being more susceptible to herbivore attack than non-woody taxa [26]. A number of studies on tropical systems have demonstrated that the extent of leaf damage may have a substantial impact on plant growth [27, 28, 29] and mortality [30]. Tree fitness is not only impaired by reduction of photosynthetic capacity [31], but also by the increased risk of viral or fungal infections through wounded plant tissues [32]. The degree to which trees are susceptible to herbivores is—among other factors—a function of tree species and surrounding environment. Tree species are thought to fall along a tolerance/resistance continuum regarding their strategic response to herbivore damage: Pioneer species tend to be characterized by a higher tolerance to herbivory and can sustain higher levels of damage without repercussions in fitness; late successional species, on the other hand, typically show greater resistance to herbivores in the form of phytochemical and physical deterrents [33, 34].

With regard to surrounding habitat, folivory is to a large part mediated by vegetational and structural diversity, but the directionality of the effect varies with intensity of management. Among the mono- and oligocultures of commercial forestry, a reduction of leaf damage with increasing plant diversity is a relatively common finding [35]. It is, however, typically related to a small number of prolific herbivore species, and highly contingent on the nature of tree species involved [36], as well as the structural characteristics of surrounding vegetation ([37, 38, 39], [40] for comprehensive review). Depending on the underlying dynamics, beneficial effects of stand diversification can be restricted to individual tree species, but absent in others [29], or overall foliar damage may even increase with plant species richness [41]. Low-intensity reforestation and natural succession, on the other hand, are likely to show an increase in herbivory with plant species richness [42] due to the concomitant rise in herbivore diversity and abundance [43].

We studied herbivory among experimental reforestation sites in the montane rainforest zone of southern Ecuador, a global biodiversity hotspot [44]. Reforestation sites have been established as part of an interdisciplinary research effort (summarized in: [45, 46]). Focal species were chosen among indigenous tree taxa based on timber value (*Cedrela montana* and *Tabebuia chrysantha*) or prospective growth performance (*Heliocarpus americanus*). Specifically, we assessed foliar damage in 6- to 7-year old trees planted among the dominant anthropogenic habitat types (i.e. pasture land, secondary shrub vegetation, and exotic timber plantations), as well as in conspecific specimens growing in the understory of natural rainforest. Sample sites thus spanned a gradient of structural complexity and plant species richness, from natural, closed-canopy forest to recently abandoned and reforested pasture land.

Specifically, we tested the following hypotheses: (1) Trees of the pioneer *H*. *americanus* experience a higher level of leaf damage compared to two later successional species, since the latter are presumed to invest more resources into anti-herbivore defence, and (2) Folivory decreases along a gradient of structural complexity and plant species richness from pasture sites to natural forest due to more effective biocontrol in increasingly diverse habitat. Furthermore, we examined temporal dynamics of leaf damage throughout the season.

## Materials and Methods

### Study area

**Fig 1:**
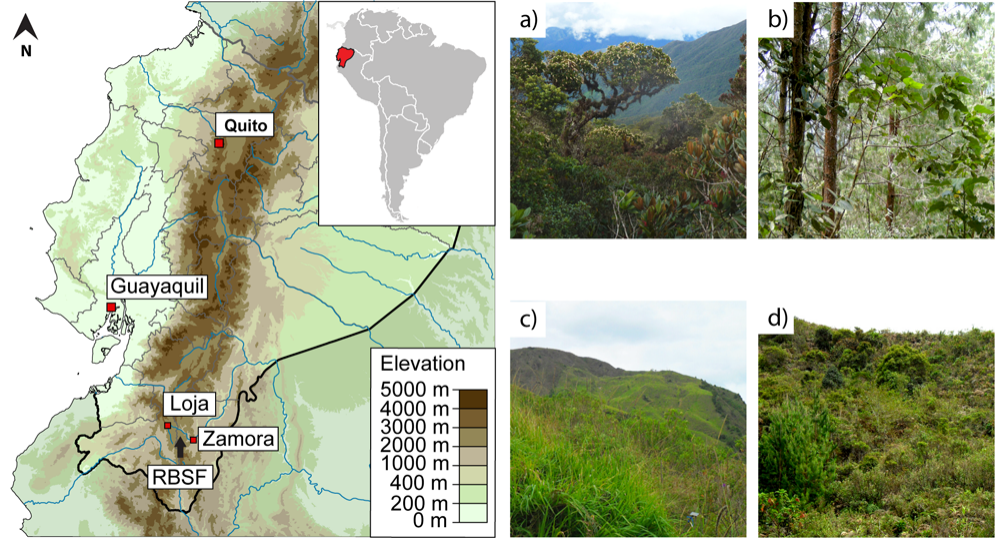
Geographic location of the Reserva Biológica San Francisco (RBSF; left panel) and prevailing vegetation types in the surrounding area (right). The images depict (a) near-natural montane rainforest, (b) commercial timber plantation (i.e. *P*. *patula*), (c) abandoned cattle pasture, and (d) secondary fern-shrub vegetation. The map is available under Creative Commons License and was modified by M.-O. Adams.

The study was conducted in and around the Reserva Biológica San Francisco (RBSF, 3°58’18”S, 79°4’45”W, 1800–2200 m a.s.l., Fig 1), located in Zamora-Chinchipe province, southern Ecuador [47, 48]. The original vegetation of the San Francisco Valley (i.e. montane rainforest; [49]) is largely conserved along the southern slopes, where the RBSF borders on the Parque Nacional Podocarpus. The opposing hillsides have been converted to agricultural use (mainly cattle farming) and currently form a patchwork of active pastures, successional shrub habitat, exotic timber plantations and small pockets of remnant ravine forest. The region is characterized by a wet season between April and July and moderately dry conditions from September to December (average annual precipitation: ~2,200 mm). Temperatures show a daily amplitude of approximately 11.1°C with an annual mean of 15.3 (± 1.2 SD)°C [50].

### Sample trees and study sites

The present study is based on experimental reforestation sites established in 2003/2004 as part of a silvicultural project. Trees were raised from local seed stock and planted among the prevalent land cover types of the region ([51, 52]; Fig 1), namely an exotic timber plantation (i.e. 25–30 year old *Pinus patula* stand, subsequently referred to as ‘Pinus’), a recently abandoned pasture still dominated by the exotic grass *Setaria sphacelata* (‘Pasture’) and mid-successional vegetation characterized by shrubs and *Pteridium arachnoideum* fern thickets (‘Shrub’; cleared ~50 years ago and undergoing unregulated secondary succession for ~25 years since the last fire). The reforestation scheme on pasture and shrub sites consisted of randomly distributed single- and mixed-species plots, each measuring 10.8 m × 10.8 m and containing 25 trees. Within the pine stand, trees were planted in eight blocks of nine 4.0 m × 4.0 m subplots. Each subplot contained a different tree species with nine individuals. On all reforestation plots, trees were evenly spaced along a grid with 1.8 m between neighbors (for details see [52]). From among the native tree species tested in these experimental plantations, we selected two deciduous, high value, mid-successional timber species (Meliaceae: *Cedrela montana* and Bignoniaceae: *Tabebuia chrysantha*), as well as one fast-growing, light-demanding, ever-green, early-successional species (Malvaceae: *Heliocarpus americanus*). Uneven survival and growth of specimens precluded a random selection of sample trees, and choice was based chiefly on foliar volume (leaf area greater than 0.15 m^2^), accessibility, and health (no excessive discoloration or wilting). Reforestation trees were compared to conspecifics from the natural regeneration pool in adjacent, unmanaged rainforest (‘Forest’), chosen to match the planted specimens in height and foliage volume. Due to high mortality of planted *H*. *americanus* within the pine stand, additional trees were similarly selected from spontaneously established stock under pine cover to increase the available sample size. Presumably due to greater sensitivity to insufficient canopy cover [53] or poor nutritional quality of the soil [54], survival and growth of *C*. *montana* on the shrub site was exceedingly low. Consequently, this tree species could not be included for this habitat type. Although spatial clumping could not always be avoided, an effort was made to assure an even and representative distribution of sample trees across sites.

A total of 160 sample trees were selected across the four habitats with an average height of 2.29 m (± 0.91 SD). Any imbalance in sample design is the result of restrictions posed by the availability of suitable trees (S1 Table). Due to the scope of the original forestry experiment, as well as the lack of viable trees and alternative sites in the surrounding landscape, the replicates for each habitat were spatially clustered. Some apparent habitat effects may therefore be idiosyncratic to the specific research sites in question, rather than representative of a broader pattern, but the major trends uncovered here deserve further exploration.

### Data collection

Since the two deciduous species among the sample trees do not bear leaves between June and September [55], surveys were conducted in six-week intervals between October 2010 and May 2011, resulting in a total of five surveys per individual tree. Assessment of folivore damage was based on ten leaves per tree and survey. To assure random choice, we successively counted leaves along the major branches and selected every *n*th leaf, where *n* was adjusted according to the overall foliar volume of the tree in question to assure an even sampling of the crown. Since destructive sampling would have interfered with ongoing silvicultural studies, leaves were recorded *in situ* using a digital camera (Panasonic Lumix DMC-TZ10). Leaves were spread out beneath a clear acrylic cover and photographed without flash against a white background including a black 1x1 cm^2^ reference scale. During digital post-processing, overly bright reflections and shadows that obscured actual herbivore damage were corrected manually with the tools available in Adobe Photoshop CS4 before assessing remnant leaf area using ImageJ (Version 1.48; [56]). In case of peripheral damage, the original area of the leaf was approximated by manually completing its contour. Based on these values, folivory was calculated as the percentage of leaf area lost to herbivore activity. For subsequent analysis, we distinguished the characteristic, semi-circular incisions of leafcutter ants (“*ant damage*”) from the varying, non-descript feeding patterns of folivorous insects (“*general damage*”). Where both kinds of damage were present, each leaf was classified according to the more prevalent type.

In addition to folivory, we recorded total leaf area per tree and survey based on leaf count of the tree in question and average leaf size at the time of survey. Similarity in the extent of foliar damage between sample trees may increase with spatial proximity due to the colonization and foraging behavior of herbivorous insects. We therefore calculated the mean distance between each tree and its five closest neighbors on the basis of GPS coordinates and included the measure as predictor in our statistical models. Lastly, tree height from ground level to the tip of the highest branch was assessed once at the beginning of the study.

Ambient temperature and humidity were recorded 1.5 m above ground at one location per habitat. Respective recording sites were chosen to be representative of the surrounding topography and vegetation structure. Readings were taken throughout the day at 30min intervals from March to September 2011 using an automated data-logger (EL-USB-2, Lascar Electronics).

A research permit for the area in question was obtained from the Ministerio del Ambiente, Ecuador.

### Statistical analysis

Folivory in the strict sense and damage related to the activity of leafcutter ants were examined independently. With regard to the former, it was necessary to sub-set the data due to the lack of *C*. *montana* on the secondary shrub site: evaluation of potential tree species effects was based on all three tree species, but excluded ‘Shrub’ habitat; conversely, habitat-specific leaf damage was estimated for all four habitat types, but only on the basis of the two shared species (*H*. *americanus* and *T*. *chrysantha*). For each tree and survey, the fraction of folivory was calculated by dividing cumulative damage by the total original surface area across the sampled leaves. Statistical evaluation was done in the R environment [57] using a generalized linear mixed model (GLMM) framework for repeated measures as implemented in the lme4 package [58]. Sample tree identity was included as random term in all models to account for non-independence of consecutive surveys on the same tree. Following the recommendation of Warton and Hui [59], leaf damage proportions were logit transformed. To account for zeros, the smallest non-zero response was added to all values. Continuous predictor variables were variously transformed using cubic-root (i.e. height), or logarithm (i.e. leaf area and distance to neighbors) to meet normality assumptions and then standardized. Models contained the primary predictor variables (habitat, tree species and time of survey), background variables (leaf area, sample tree height, and distance to nearest neighbor trees), all two-way interactions between the primary predictor variables as well as between habitat and the background variables, and the three-way interaction between habitat, tree species and time of survey. Goodness of fit of the best model was expressed as marginal (variance explained by fixed factors) and conditional (variance explained by fixed and random factors) R^2^, following the method of Nakagawa and Schielzeth [60].

Ant-related damage was likewise aggregated by sample tree and survey and expressed as percentage relative to the corresponding total surface area of sampled leaves (disregarding any non-ant herbivory). Due to the clear bias of leafcutter ants towards *T*. *chrysantha* on pasture- and shrub sites, subsequent analysis was restricted to these data points. Habitat-specific distributions were compared by a two sample Cramér-von Mises test using functions from the R packages CDFt [61] and CvM2SL2Test [62], respectively.

## Results

Over the course of the study, we sampled a total of 7,290 leaves across three tree species and four habitats. The average level of leaf damage due to herbivore activity was relatively low throughout the study. Nonetheless, analyses indicated several strong predictors of folivore damage, namely surrounding habitat, sample tree species, date of survey, and various interactions thereof (Table 1).

**Table 1:** Results of GLMM analyses on proportional leaf area damage across all surveys with sample tree identity as random term. Leaves damaged by leaf-cutter ants were excluded. Calculations are based on data subsets (a) across all four habitats but excluding *C*. *montana* and (b) across all three tree species but excluding ‘Shrub’ habitat. Goodness of fit is expressed as Nakagawa’s marginal and conditional R^2^.

	(a)			(b)		
	df	F	p	df	F	p
**Habitat**	3	10.604	< 0.001***	2	4.903	0.009**
**Tree species**	1	6.570	0.012*	2	31.765	< 0.001***
**Survey number**	4	7.165	< 0.001***	4	5.378	< 0.001***
**Total leaf area**	1	0.504	0.478	1	3.942	0.048*
**Height**	1	0.087	0.769	1	0.078	0.781
**Dist. to neighbors**	1	0.134	0.715	1	0.127	0.722
**Habitat × Tree sp.**	3	2.185	0.094.	4	10.897	< 0.001***
**Habitat × Height**	3	0.379	0.769	2	3.337	0.039*
**Tree sp. × Survey nr.**	4	6.879	< 0.001***	8	5.370	< 0.001***
**Habitat × Survey nr.**	12	1.944	0.028*	8	1.387	0.200
**Habitat × Dist. to neighbors**	3	0.946	0.421	2	0.162	0.851
**Habitat × Tree sp. × Survey nr.**	12	2.552	0.003**	16	1.947	0.015*
**Nakagawa’s marginal R**^**2**^		0.3036			0.4345	
**Nakagawa’s conditional R**^**2**^		0.5806			0.6671	

Significance codes

p ≤ 0.001 ‘***’

p ≤ 0.01 ‘**’

p ≤ 0.05 ‘*’

p ≤ 0.1 ‘.’

**Fig 2:**
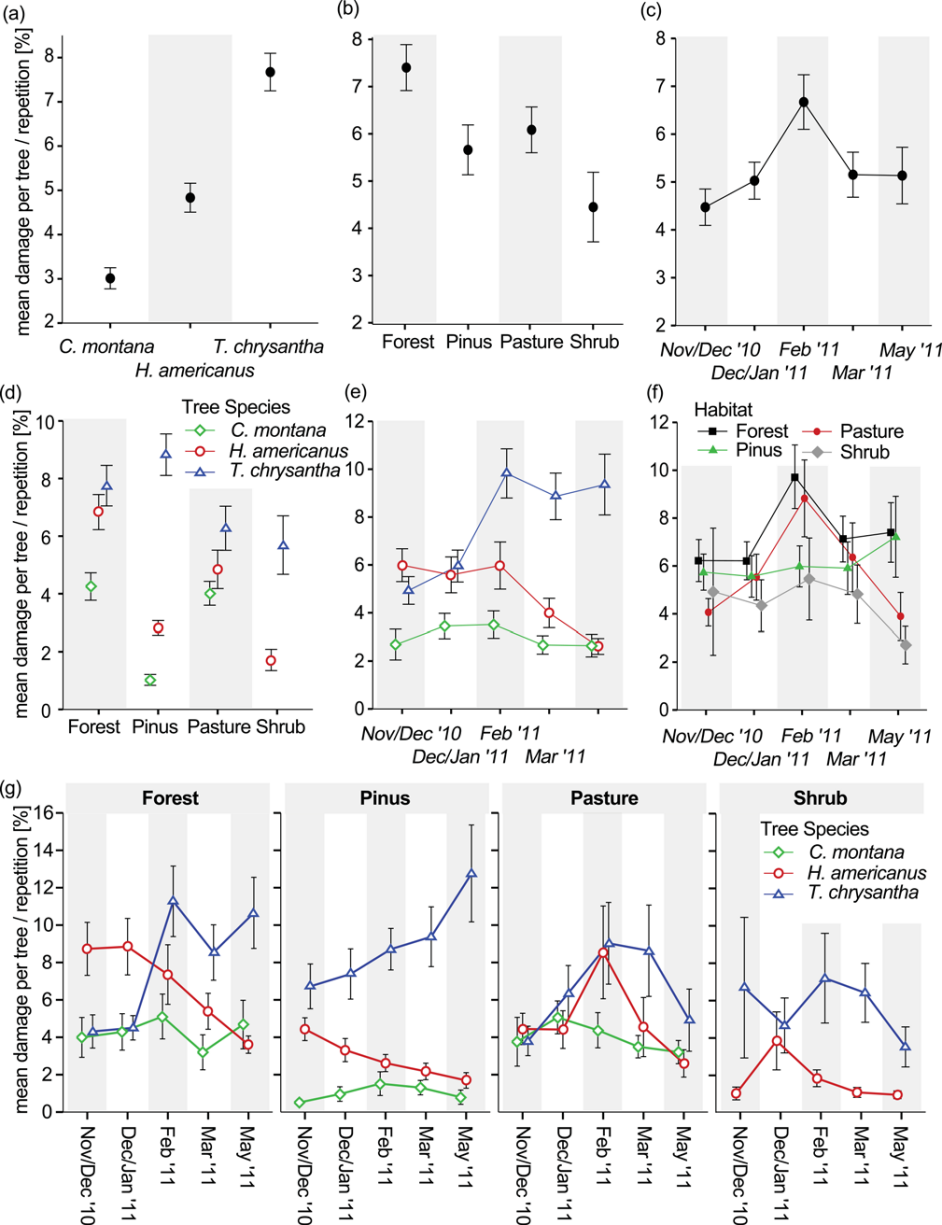
**Relative leaf damage (in percent) as a function of (a) tree species, (b) habitat, (c) survey, (d) tree sp. × habitat, (e) tree sp. × survey, (f) habitat × survey and (g) tree sp. × habitat × survey**. Due to the imbalance in the dataset, analyses had to be based on two different data subsets. In diagram (a) and (f) mean damage was calculated for *H*. *americanus* and *T*. *chrysantha* only, excluding *C*. *montana*. For diagrams (b), (c), and (e), ‘Shrub’ sites were excluded prior to aggregation. Graphics (d) and (g) are based on the complete dataset to facilitate comparison. Points represent mean proportional leaf damage and whiskers the corresponding standard error of the mean.

Leaf area loss was significantly higher in *T*. *chrysantha* (7.7% ± 0.4 standard error), compared to *H*. *americanus* (4.8% ± 0.3 SE), which in turn suffered more damage than *C*. *montana* (3.4% ± 0.2 SE; Fig 2A). The habitat effect was likewise significant, with the highest level of folivory in undisturbed forest, intermediate damage in both pasture and pine plantation, and lowest values found for trees among successional shrub vegetation (Fig 2B). The peak in average leaf area loss around February (Fig 2C) arises from the overlap of underlying, but quite distinct habitat- and species-specific patterns (discussed below). Herbivory on individual tree species was significantly contingent on the habitat. Relative to the ‘Forest’ and ‘Pasture’ sites, trees planted beneath *P*. *patula* were characterized by higher proportional leaf damage in *T*. *chrysantha*, but lower herbivory in both *C*. *montana* and *H*. *americanus*. (Fig 2D). The patterns of leaf damage across survey events as a function of tree species (Fig 2E) or habitat (Fig 2F), respectively, are best interpreted in light of the underlying and likewise significant three-way interaction (Fig 2G). Throughout the recording period, *C*. *montana* maintained a fairly consistent level of herbivory across all habitats. *H*. *americanus* trees beneath canopy cover experienced a steady decrease in folivory over time, while specimens on open habitat showed a peak in herbivory around January/February. In *T*. *chrysantha*, leaf damage accumulated steadily beneath *P*. *patula* and asymptotically in natural forest. The unimodal trajectory among trees on open sites (Pasture and Shrub) may be related to the intermittent flush of fresh foliage in individual sample trees (S2 Table), leading to an apparent decline in leaf damage. Other environmental predictor variables, including total leaf area, height, proximity to neighboring sample trees, and their respective interaction terms had no or only comparatively weak effects on herbivore damage (Table 1).

**Fig 3:**
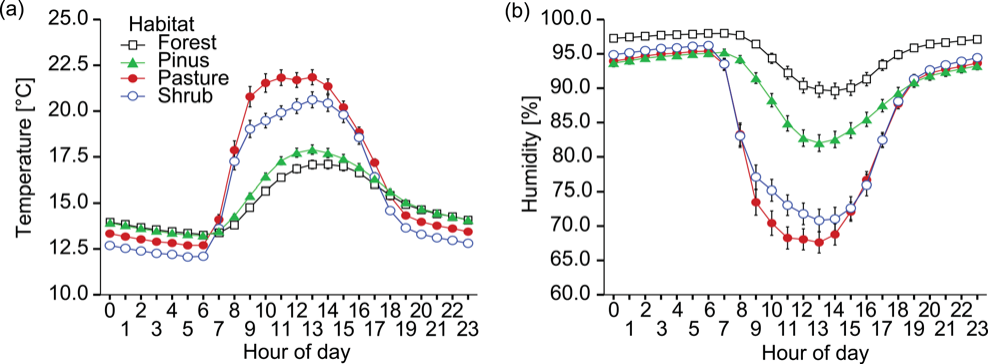
**Hourly means for (a) temperature and (b) humidity as a function of habitat**. Measurements were recorded 1.5m above ground at intervals of 30 minutes from March to September, 2011. Graphs show means (± standard error of the mean).

Microclimatic conditions varied markedly between habitats. On average, noon-time temperatures were 3–5°C higher on reforestation sites without canopy cover, compared to forested sites. Furthermore, open habitat was characterized by a steeper rise in temperature during the morning hours. Humidity showed a complementary pattern with considerably lower midday values for ‘Pasture’ and ‘Shrub’ sites, relative to the *P*. *patula* plantation and undisturbed forest (Fig 3).

Leaf area loss due to harvesting activity of leafcutter ants was clearly biased towards trees of *T*. *chrysantha* growing on more open habitat. Among secondary shrub habitat, 52.6% of respective sample trees exhibited signs of leafcutter ant foraging activity at least once during the course of the study, with 24.0% of respective leaf samples showing ant-related damage. By comparison, on pasture sites, only 7 out of 18 *T*. *chrysantha* trees (38.9%) and 5.2% of associated sample leaves showed signs of ant harvesting activity. On average, leaf area loss due to leafcutter ants per tree and survey was significantly lower for *T*. *chrysantha* on pasture sites (Pasture: 2.5 ± 12.0% SD; Shrub: 10.5 ± 17.2% SD; T = 3.250, p < 0.001). In both habitats, ant damage to individual trees could nonetheless be extensive with losses of up to 92.5% of total leaf area in pasture sites and 92.8% for sample trees among secondary shrub vegetation. Intermittent defoliation followed by a fresh flush of foliage was most prevalent among *T*. *chrysantha* trees planted in ‘Pasture’ and ‘Shrub’ sites. With regard to other habitats and plant species, the phenomenon was rare and restricted to single plant individuals (S2 Table). Since there were no remnant petioles or visible traces ant activity, the ultimate cause of defoliation could not be determined with certainty. Harvest by leafcutter ants remains the most likely explanation, however.

## Discussion

Based on a sample of more than 7,000 leaves measured over a complete growing season, the present study examined the extent of insect folivory on native tree species across different reforestation sites in the tropical Andes relative to undisturbed rainforest. We distinguished between herbivory in the strict sense (i.e. primarily caused by Lepidoptera larvae and Coleoptera; [63]) and foraging activity of leafcutter ants, which represent key herbivores and significant pests in Neotropical ecosystems [64, 65].

Overall folivory by insects other than ants was low with an average leaf area loss of 3.2–7.5% depending on tree species. While this is considerably below the values typically cited for tropical rainforests (i.e. 11.1% for shade-tolerant understory species and 48.0% for gap specialists in lowlands; [66]), a recent meta-analysis suggests that the extent of tropical herbivory may have been overestimated in the past [26, 67]. Host-specific folivory in the present case resembled the findings for congeneric species at other tropical plantation sites (e.g. *Tabebuia rosea*: [29], *T*. *ochracea*: [68]), and was frequently lower by comparison (*Heliocarpus pallidus*: [69], *Cedrela odorata*: [70], e.g. *Tabebuia aurea* and *T*. *ochracea*: [71]). In part, this probably reflects altitudinal effects, since herbivory generally declines with increasing elevation [72]. Generally speaking, leaf area loss due to insect herbivory therefore does not seem to constitute a major limiting factor for reforestation on abandoned pasture sites in the montane forest zone around 2,000 m elevation. Nonetheless, we observed significant differences between groups of sampled trees, with tree species, surrounding habitat, date of survey and the respective two- and three-way interactions thereof emerging as the primary predictive variables.

Contrary to our initial expectations based on the differentiation between damage-tolerant pioneer species and defense-oriented, successional taxa [73], folivory was highest in *T*. *chrysantha* followed by *H*. *americanus* and *C*. *montana* across reforestation sites as well as in natural forest. Although phytochemical composition was not assessed in the present study, the observed pattern may have been due to general palatability of the tree species in question: Members of the Meliaceae family are known to employ potent secondary metabolites to deter herbivores [74], and *H*. *americanus* has been shown to contain defensive compounds (e.g. phenolics, terpenes and tannines) at higher concentrations than a congeneric of *T*. *chrysantha* (*Tabebuia rosea*; [75]). Furthermore, any gradient in herbivore damage between pioneer and successional species is inevitably affected by the superimposed distinction between ever-green and deciduous taxa in the present study. Pringle *et al*. [76] found that evergreen trees tended to experience lower rates of herbivory, and postulated that the traits distinguishing deciduous and evergreen species are similar to those differentiating between light-demanding and shade-tolerant species, thus leading to a convergence of defensive syndromes. While such chemical countermeasures may have limited effect on host-specialists, they can be expected to repel a substantial number of generalist or accidental consumers. In the present study, assemblages of chewing herbivores were mostly dominated by a large number of species with low individual abundance [63], suggesting that prolific specialists were rare (except in pine sites; see below).

In addition to phytochemical traits, other defensive measures may have played a role in the present context. Unlike *C*. *montana*, *T*. *chrysantha* and possibly also *H*. *americanus* possess extra-floral nectaries (personal observation), which implicate ant mutualism as part of their defensive strategy. Such mutualistic ant-plant relations are generally beneficial [77] and can be as effective as phytochemical defense [78], but the degree of protection is contingent on the presence of suitable ant species (e.g. [79, 80]). Abundance and species richness of ants typically decline with altitude [81, 82], and ant-based defensive strategies may therefore become increasingly ineffective with elevation.

Likewise contrary to our initial assumption, leaf damage was highest in undisturbed forest as opposed to reforestation sites. This discrepancy between expectations and observed pattern was mirrored by a parallel increase in abundance and diversity of phytophagous insects [63]. This corresponds to findings in tropical forests [43] and temperate-zone grassland systems [83, 84, 85, 86], which likewise demonstrated higher density and diversity of herbivores with greater plant species richness, accompanied by a concurrent increase in herbivory. With regard to silvicultural settings, the often cited inverse correlation between stand diversification and herbivory on which our initial prediction was based, seems to be more typical for inherently species-poor plantation systems ([35] and citations therein). More natural forest stands, on the other hand, often seem to show increased levels of folivory with greater tree species richness (e.g. [42]).

At first glance, reforestation sites seem to adhere to the abovementioned reduction of herbivory with increased stand diversity: the successional shrub site was characterized by substantially higher plant species richness than either pasture or pine habitat ([87]; J. Gawlik, unpublished data), and showed correspondingly lower levels of foliar damage. An overall sparsity of insects and arachnids on the shrub habitat [63, 88], however, suggests that the observed pattern in herbivore damage is at least partly a function of microclimate, rather than of floristic diversity or increased activity of natural enemies. Folivory has been shown to decrease at high levels of insolation due to alterations in host-plant phytochemistry and physiology [89, 90, 91]. Associated changes in water availability likewise affect palatability and nutritional value of prospective host plants [92]. This latter aspect might account for the discrepancy between the two open reforestation sites (i.e. Pasture and Shrub). Our climate measurements were taken at 1.5m above ground and may not adequately represent conditions at ground level. Shrub habitat showed a high percentage of bare ground between sample trees and may in consequence loose water more quickly. Pastures, on the other hand, maintained a dense cover of grass (*Setaria sphacelata*), which may play a role in maintaining soil water content or increasing precipitation by filtering moisture from fog [93]. Some herbivores (e.g. Acrididae) are known to favor dry, open habitat, but these species appeared to play only a negligible role in the present context. The two major herbivore orders in reforestation sites according to our data (i.e. Coleoptera and Lepidoptera) mirrored the pattern of leaf damage reported here, with abundance and species richness declining along the proposed microclimatic gradient [63]. Based on the present data, however, it is difficult to disentangle whether the observed differences in herbivory are related to aspects of host plant quality or due to insects avoiding unfavorable microclimatic conditions.

Ultimately, potential benefits for young trees derived from slightly lower herbivore pressure on the successional shrub site are likely to be offset by reduced survival and growth due to harsher abiotic conditions. Especially shade-adapted mid- to late-successional timber species such as *C*. *montana* and *T*. *chrysantha* showed reduced performance at a canopy openness above 30% [53].

Folivory among enrichment plantings beneath *P*. *patula* was predominantly focused on *T*. *chrysantha*, highlighting the point that habitat effects vary depending on plant species. Pine plantations in the Andean highlands are often established on poor soil and can be subject to severe cation deficiencies (Mg^++^, Ca^++^, and K^+^: [94, 95]). *T*. *chrysantha* may be more sensitive to unfavorable soil conditions compared to the other two sample species, thus rendering it more susceptible to herbivore attack. Alternatively, conditions beneath *P*. *patula* may favor certain phytophagous taxa that feed preferentially on *T*. *chrysantha*. Two fairly abundant species of leaf beetles (Chrysomelidae: Galerucinae spp.) and two species of Lepidoptera (Gelechiidae sp. and Bombycidae: Bombycinae sp.) showed notably high densities on pine sites and fed almost exclusively on *T*. *chrysantha* [88]. Cooler conditions beneath canopy have been linked to higher *per capita* consumption rates in herbivorous insects [96], and may have further aggravated the impact of these species.

In interpreting the habitat effects outlined above, it is important to note that the observed folivory patterns cannot be unconditionally attributed to the respective habitat types, since sample sites were by necessity clustered. Reforestation sites may have inadvertently differed with regard to other aspects (e.g. soil conditions, exposition, topography) that could have conceivably influenced folivory. Given the sparsity of data on herbivory in tropical highland silviculture, the present study nonetheless offers important insights that merit closer examination in future studies.

Progression of folivory throughout the survey period varied between sample tree species. Individuals of the deciduous *C*. *montana* suffered herbivore attack during the flush of fresh foliage at the beginning of the dry season (compare [66]), but experienced little subsequent damage regardless of surrounding habitat. Representatives of the likewise deciduous *T*. *chrysantha* were subject to fairly constant herbivore pressure up to the middle of the dry season in February. After this point, damage rates declined in both undisturbed rainforest and reforestation sites, with the notable exception of enrichment plantings beneath *P*. *patula*. *H*. *americanus* on open reforestation sites (Pasture and Shrub) showed a conspicuous peak in herbivory in January and February which was absent in forested sites. The cause is difficult to discern, as neither Coleoptera nor Lepidoptera showed a noticeably increase in abundance during the time in question [88]. A possible candidate are Orthopterans, which were present in small numbers on open habitat but largely absent beneath canopy cover (personal observation).

Foraging activity of leafcutter *Acromyrmex* ants was limited almost exclusively to *T*. *chrysantha* on open habitat, where they occasionally caused severe damage. Although polyphagous, leafcutter ants are highly selective in their choice of host, and tend to avoid plants containing high concentrations of secondary metabolites that might negatively affect the growth of their fungal cultivar [97, 98, 99]. Consequently, the observed host-plant preference in *Acromyrmex* sp. corresponds to the gradient of toxicity proposed above. This predisposition may have been further augmented by an affinity of leafcutter ants towards drought-stressed foliage [100, 101]. Throughout the course of the study, microclimate on open habitat was considerably warmer and dryer during daytime compared to forested sites. Under such conditions, shade-adapted, late successional species like *T*. *chrysantha* may experience water-stress sooner than light-seeking pioneers, thus rendering such trees more susceptible to ant attack. The restriction of *Acromyrmex* activity to habitat without canopy cover is likely due to the narrow thermal optima of their symbiotic fungi. The geographic distribution of leafcutter ants is typically restricted to altitudes below 2,000 m a.s.l. ([65] and citations therein, [102]), indicating that our research area was located close to the upper limit of the species’ distribution. Ambient temperatures on secondary shrub and pasture sites increased more rapidly in the morning and reached higher day-time averages, apparently allowing successful colonization and subsequent persistence of leafcutter ants in open habitat, but effectively excluding them from forested sites (compare [103]). A tendency towards greater activity and presumably abundance of *Acromyrmex* ants on successional shrub sites is likely connected to the lack of insulating ground cover, which can be expected to coincide with higher maximum surface and soil temperatures.

In summary, the relatively low levels of leaf damage observed in the study area suggest that insect herbivory is currently not a limiting factor for targeted reforestation in the equatorial Andean highlands. Leafcutter ants of the genus *Acromyrmex* are a notable exception in this context, since they cannot only cause extensive damage, but may also selectively target high-value timber species (i.e. *T*. *chrysantha*). While their activity is fairly limited at present, the rise in average temperatures predicted by climate change models implies a far greater pest potential in the future [104]. Nursery canopies of exotic or preferentially native tree species may offer a cost effective way to control leafcutter ants. The autochthonous pioneer *H*. *americanus* is a promising candidate in this regard, due to its high growth rate and comparatively low apparent attractiveness to leaf-cutter ants and other potential herbivores.

## Supporting Information

S1 Table GPS coordinates of sample trees. Where appropriate, affiliation with pre-existing experimental plots is given. Plot identifiers are in accordance with the original work of Aguirre [52]. Due to a lack of planted saplings, sample trees in undisturbed forest and *H*. *americanus* trees in the pine plantation were recruited from natural regeneration stock.
(DOCX)

S2 Table Overview of the number of trees per survey characterized by a fresh flush of foliage after being leafless in the previous recording. The numerals before and after the slash denote the number of trees exhibiting fresh leaves, and the total number of foliage-bearing trees, respectively. The corresponding percentage value is given in parentheses. The first survey is not taken into account since both deciduous species (i.e. *C*. *montana* and *T*. *chrysantha*) typically develop new foliage during this time.
(DOCX)

S3 Table Raw data. (CSV)
