# Supplementary material for: Low Herbivory among Targeted Reforestation Sites in the Andean Highlands of Southern Ecuador
Source: PLoS One. 2016 Mar 10;11(3):e0151277. doi: 10.1371/journal.pone.0151277 (PMC4786223; doi:10.1371/journal.pone.0151277)
Supplement: S1 Table — Where appropriate, affiliation with pre-existing experimental plots is given. Plot identifiers are in accordance with the original work of Aguirre [52]. Due to a lack of planted saplings, sample trees in undisturbed forest and H. americanus trees in the pine plantation were recruited from natural regeneration stock. (DOCX) [file pone.0151277.s001.docx]

| **Habitat** | **Species** | | **Tree ID** | **Altitude [m]** | **GPS position [UTM]** | **Plot ID*** | **Plot type*** |
| --- | --- | --- | --- | --- | --- | --- | --- |
| Forest | *Cedrela montana* | | cm01 | 1893 | 17 M 0713129 9560130 | n.a. | natural regeneration |
| Forest | *Cedrela montana* | | cm02 | 2028 | 17 M 0714274 9560413 | n.a. | natural regeneration |
| Forest | *Cedrela montana* | | cm03 | 1943 | 17 M 0714246 9560509 | n.a. | natural regeneration |
| Forest | *Cedrela montana* | | cm04 | 1965 | 17 M 0714243 9560452 | n.a. | natural regeneration |
| Forest | *Cedrela montana* | | cm05 | 1996 | 17 M 0714271 9560409 | n.a. | natural regeneration |
| Forest | *Cedrela montana* | | cm06 | 1994 | 17 M 0714273 9560419 | n.a. | natural regeneration |
| Forest | *Cedrela montana* | | cm07 | 1813 | 17 M 0713292 9560477 | n.a. | natural regeneration |
| Forest | *Cedrela montana* | | cm08 | 1824 | 17 M 0713248 9560408 | n.a. | natural regeneration |
| Forest | *Cedrela montana* | | cm09 | 1905 | 17 M 0713886 9560640 | n.a. | natural regeneration |
| Forest | *Cedrela montana* | | cm10 | 2000 | 17 M 0713944 9560480 | n.a. | natural regeneration |
| Forest | *Heliocarpus americanus* | | ha01 | 1897 | 17 M 0713145 9560142 | n.a. | natural regeneration |
| Forest | *Heliocarpus americanus* | | ha02 | 1830 | 17 M 0713350 9560570 | n.a. | natural regeneration |
| Forest | *Heliocarpus americanus* | | ha03 | 1879 | 17 M 0712855 9560070 | n.a. | natural regeneration |
| Forest | *Heliocarpus americanus* | | ha04 | 1969 | 17 M 0713964 9560520 | n.a. | natural regeneration |
| Forest | *Heliocarpus americanus* | | ha05 | 1830 | 17 M 0713350 9560570 | n.a. | natural regeneration |
| Forest | *Heliocarpus americanus* | | ha06 | 1830 | 17 M 0713349 9560560 | n.a. | natural regeneration |
| Forest | *Heliocarpus americanus* | | ha07 | 1912 | 17 M 0713481 9560531 | n.a. | natural regeneration |
| Forest | *Heliocarpus americanus* | | ha08 | 1948 | 17 M 0713055 9560060 | n.a. | natural regeneration |
| Forest | *Heliocarpus americanus* | | ha09 | 1955 | 17 M 0713041 9560020 | n.a. | natural regeneration |
| Forest | *Heliocarpus americanus* | | ha10 | 1967 | 17 M 0713044 9560000 | n.a. | natural regeneration |
| Forest | *Heliocarpus americanus* | | ha12 | 1979 | 17 M 0714145 9560403 | n.a. | natural regeneration |
| Forest | *Heliocarpus americanus* | | ha17 | 1994 | 17 M 0714275 9560409 | n.a. | natural regeneration |
| Forest | *Heliocarpus americanus* | | ha20 | 1976 | 17 M 0714278 9560434 | n.a. | natural regeneration |
| Forest | *Tabebuia chrysantha* | | tc02 | 1872 | 17 M 0713439 9560505 | n.a. | natural regeneration |
| Forest | *Tabebuia chrysantha* | | tc03 | 1840 | 17 M 0712989 9560210 | n.a. | natural regeneration |
| Forest | *Tabebuia chrysantha* | | tc04 | 1833 | 17 M 0712996 9560207 | n.a. | natural regeneration |
| Forest | *Tabebuia chrysantha* | | tc05 | 1884 | 17 M 0713437 9560490 | n.a. | natural regeneration |
| Forest | *Tabebuia chrysantha* | | tc06 | 1875 | 17 M 0713456 9560494 | n.a. | natural regeneration |
| Forest | *Tabebuia chrysantha* | | tc07 | 1919 | 17 M 0713153 9560177 | n.a. | natural regeneration |
| Forest | *Tabebuia chrysantha* | | tc08 | 1962 | 17 M 0714236 9560470 | n.a. | natural regeneration |
| Forest | *Tabebuia chrysantha* | | tc09 | 1878 | 17 M 0713158 9560169 | n.a. | natural regeneration |
| Forest | *Tabebuia chrysantha* | | tc10 | 2012 | 17 M 0714141 9560404 | n.a. | natural regeneration |
| Forest | *Tabebuia chrysantha* | | tc11 | 2006 | 17 M 0714282 9560402 | n.a. | natural regeneration |
| Forest | *Tabebuia chrysantha* | | tc12 | 1999 | 17 M 0714282 9560399 | n.a. | natural regeneration |
| Forest | *Tabebuia chrysantha* | | tc13 | 1831 | 17 M 0713350 9560560 | n.a. | natural regeneration |
| Forest | *Tabebuia chrysantha* | | tc14 | 1873 | 17 M 0713449 9560492 | n.a. | natural regeneration |
| Forest | *Tabebuia chrysantha* | | tc15 | 2009 | 17 M 0714295 9560402 | n.a. | natural regeneration |
| Forest | *Tabebuia chrysantha* | | tc16 | 1813 | 17 M 0713242 9560412 | n.a. | natural regeneration |
| Forest | *Tabebuia chrysantha* | | tc17 | 1964 | 17 M 0713974 9560530 | n.a. | natural regeneration |
| Forest | *Tabebuia chrysantha* | | tc18 | 1890 | 17 M 0713145 9560171 | n.a. | natural regeneration |
| Forest | *Tabebuia chrysantha* | | tc19 | 1830 | 17 M 0712975 9560191 | n.a. | natural regeneration |
| Forest | *Tabebuia chrysantha* | | tc20 | 1988 | 17 M 0714141 9560395 | n.a. | natural regeneration |
| Forest | *Tabebuia chrysantha* | | tc21 | 2014 | 17 M 0714143 9560408 | n.a. | natural regeneration |
| Pasture | *Cedrela montana* | | cm01 | 1955 | 17 M 0713465 9561024 | 120 | Single species |
| Pasture | *Cedrela montana* | | cm02 | 1924 | 17 M 0713538 9560968 | 155 | Mixed species ^A^ |
| Pasture | *Cedrela montana* | | cm03 | 1922 | 17 M 0713545 9560967 | 155 | Mixed species ^A^ |
| Pasture | *Cedrela montana* | | cm04 | 1932 | 17 M 0713523 9560977 | 143 | Single species |
| Pasture | *Cedrela montana* | | cm05 | 1947 | 17 M 0713480 9561004 | 123 | Single species |
| Pasture | *Cedrela montana* | | cm06 | 1932 | 17 M 0713523 9560990 | 140 | Mixed species ^A^ |
| Pasture | *Cedrela montana* | | cm07 | 1947 | 17 M 0713484 9561008 | 128 | Mixed species ^A^ |
| Pasture | *Cedrela montana* | | cm08 | 1947 | 17 M 0713490 9561006 | 128 | Mixed species ^A^ |
| Pasture | *Cedrela montana* | | cm09 | 1903 | 17 M 0713553 9560907 | 180 | Single species |
| Pasture | *Cedrela montana* | | cm10 | 1901 | 17 M 0713486 9560937 | 138 | Mixed species ^A^ |
| Pasture | *Cedrela montana* | | cm11 | 1930 | 17 M 0713526 9560995 | 140 | Mixed species ^A^ |
| Pasture | *Cedrela montana* | | cm12 | 1902 | 17 M 0713556 9560904 | 180 | Single species |
| Pasture | *Cedrela montana* | | cm13 | 1912 | 17 M 0713553 9560908 | 180 | Single species |
| Pasture | *Cedrela montana* | | cm14 | 1902 | 17 M 0713559 9560911 | 180 | Single species |
| Pasture | *Cedrela montana* | | cm15 | 1947 | 17 M 0713460 9561018 | 121 | Single species |
| Pasture | *Cedrela montana* | | cm16 | 1946 | 17 M 0713481 9561003 | 123 | Single species |
| Pasture | *Cedrela montana* | | cm17 | 1945 | 17 M 0713458 9561019 | 121 | Single species |
| Pasture | *Cedrela montana* | | cm18 | 1892 | 17 M 0713500 9560926 | n.a. | n.a. |
| Pasture | *Heliocarpus americanus* | | ha01 | 1903 | 17 M 0713535 9560908 | 200 | Mixed species ^B^ |
| Pasture | *Heliocarpus americanus* | | ha02 | 1917 | 17 M 0713590 9560954 | 174 | Single species |
| Pasture | *Heliocarpus americanus* | | ha03 | 1929 | 17 M 0713583 9560953 | 171 | Single species |
| Pasture | *Heliocarpus americanus* | | ha04 | 1921 | 17 M 0713591 9560959 | 174 | Single species |
| Pasture | *Heliocarpus americanus* | | ha05 | 1924 | 17 M 0713590 9560963 | 174 | Single species |
| Pasture | *Heliocarpus americanus* | | ha06 | 1960 | 17 M 0713402 9561022 | 77 | Single species |
| Pasture | *Heliocarpus americanus* | | ha07 | 1946 | 17 M 0713336 9561015 | 35 | Single species |
| Pasture | *Heliocarpus americanus* | | ha08 | 1964 | 17 M 0713303 9561027 | 7 | Single species |
| Pasture | *Heliocarpus americanus* | | ha09 | 1954 | 17 M 0713457 9561026 | 128 | Mixed species ^A^ |
| Pasture | *Heliocarpus americanus* | | ha11 | 2072 | 17 M 0713655 9561450 | 234 | Single species |
| Pasture | *Heliocarpus americanus* | | ha13 | 1959 | 17 M 0713309 9561031 | 7 | Single species |
| Pasture | *Tabebuia chrysantha* | | tc01 | 1924 | 17 M 0713567 9560931 | 177 | Single species |
| Pasture | *Tabebuia chrysantha* | | tc02 | 1999 | 17 M 0713570 9560936 | 177 | Single species |
| Pasture | *Tabebuia chrysantha* | | tc03 | 2055 | 17 M 0713711 9561438 | 201 | Single species |
| Pasture | *Tabebuia chrysantha* | | tc04 | 2048 | 17 M 0713712 9561436 | 201 | Single species |
| Pasture | *Tabebuia chrysantha* | | tc05 | 2057 | 17 M 0713718 9561434 | 201 | Single species |
| Pasture | *Tabebuia chrysantha* | | tc06 | 2056 | 17 M 0713716 9561434 | 201 | Single species |
| Pasture | *Tabebuia chrysantha* | | tc07 | 2056 | 17 M 0713717 9561439 | 201 | Single species |
| Pasture | *Tabebuia chrysantha* | | tc08 | 2059 | 17 M 0713720 9561436 | 201 | Single species |
| Pasture | *Tabebuia chrysantha* | | tc09 | 1935 | 17 M 0713328 9560987 | 27 | Single species |
| Pasture | *Tabebuia chrysantha* | | tc10 | 1924 | 17 M 0713322 9560992 | 27 | Single species |
| Pasture | *Tabebuia chrysantha* | | tc11 | 1939 | 17 M 0713327 9560997 | 27 | Single species |
| Pasture | *Tabebuia chrysantha* | | tc12 | 1935 | 17 M 0713367 9560996 | 58 | Single species |
| Pasture | *Tabebuia chrysantha* | | tc13 | 1941 | 17 M 0713331 9560995 | 27 | Single species |
| Pasture | *Tabebuia chrysantha* | | tc14 | 1936 | 17 M 0713332 9560991 | 27 | Single species |
| Pasture | *Tabebuia chrysantha* | | tc15 | 1942 | 17 M 0713333 9560997 | 27 | Single species |
| Pasture | *Tabebuia chrysantha* | | tc16 | 1933 | 17 M 0713367 9560999 | 58 | Single species |
| Pasture | *Tabebuia chrysantha* | | tc17 | 2051 | 17 M 0713714 9561437 | 201 | Single species |
| Pasture | *Tabebuia chrysantha* | | tc18 | 1899 | 17 M 0713488 9560938 | 138 | Mixed species ^A^ |
| Pinus | *Cedrela montana* | | cm01 | 2048 | 17 M 0713274 9561163 | G4 | Species subplot ^C^ |
| Pinus | *Cedrela montana* | | cm02 | 2016 | 17 M 0713285 9561110 | G2 | Species subplot ^C^ |
| Pinus | *Cedrela montana* | | cm03 | 2032 | 17 M 0713269 9561156 | G4 | Species subplot ^C^ |
| Pinus | *Cedrela montana* | | cm04 | 2039 | 17 M 0713271 9561164 | G4 | Species subplot ^C^ |
| Pinus | *Cedrela montana* | | cm05 | 2026 | 17 M 0713235 9561096 | P3 | Species subplot ^C^ |
| Pinus | *Cedrela montana* | | cm06 | 2030 | 17 M 0713241 9561107 | P3 | Species subplot ^C^ |
| Pinus | *Cedrela montana* | | cm07 | 2028 | 17 M 0713240 9561102 | P3 | Species subplot ^C^ |
| Pinus | *Cedrela montana* | | cm09 | 2024 | 17 M 0713287 9561109 | G2 | Species subplot ^C^ |
| Pinus | *Cedrela montana* | | cm10 | 1990 | 17 M 0713320 9561079 | G1 | Species subplot ^C^ |
| Pinus | *Cedrela montana* | | cm12 | 2029 | 17 M 0713285 9561095 | P2 | Species subplot ^C^ |
| Pinus | *Cedrela montana* | | cm13 | 2010 | 17 M 0713297 9561054 | P1 | Species subplot ^C^ |
| Pinus | *Cedrela montana* | | cm14 | 1982 | 17 M 0713317 9561082 | G1 | Species subplot ^C^ |
| Pinus | *Cedrela montana* | | cm15 | 2022 | 17 M 0713293 9561113 | G2 | Species subplot ^C^ |
| Pinus | *Cedrela montana* | | cm16 | 2021 | 17 M 0713285 9561111 | G2 | Species subplot ^C^ |
| Pinus | *Cedrela montana* | | cm17 | 2031 | 17 M 0713245 9561124 | G3 | Species subplot ^C^ |
| Pinus | *Heliocarpus americanus* | | ha02 | 1994 | 17 M 0713261 9561048 | n.a. | natural regeneration |
| Pinus | *Heliocarpus americanus* | | ha03 | 1979 | 17 M 0713265 9561055 | n.a. | natural regeneration |
| Pinus | *Heliocarpus americanus* | | ha04 | 1991 | 17 M 0713261 9561047 | n.a. | natural regeneration |
| Pinus | *Heliocarpus americanus* | | ha05 | 2004 | 17 M 0713277 9561040 | n.a. | natural regeneration |
| Pinus | *Heliocarpus americanus* | | ha06 | 1983 | 17 M 0713324 9561077 | G1 | Species subplot ^C^ |
| Pinus | *Heliocarpus americanus* | | ha07 | 2002 | 17 M 0713323 9561061 | n.a. | natural regeneration |
| Pinus | *Heliocarpus americanus* | | ha08 | 1990 | 17 M 0713243 9561031 | n.a. | natural regeneration |
| Pinus | *Heliocarpus americanus* | | ha09 | 2013 | 17 M 0713262 9561082 | n.a. | natural regeneration |
| Pinus | *Heliocarpus americanus* | | ha10 | 1975 | 17 M 0713270 9561039 | n.a. | natural regeneration |
| Pinus | *Heliocarpus americanus* | | ha11 | 2042 | 17 M 0713253 9561123 | G3 | Species subplot ^C^ |
| Pinus | *Heliocarpus americanus* | | ha12 | 2040 | 17 M 0713251 9561120 | G3 | Species subplot ^C^ |
| Pinus | *Heliocarpus americanus* | | ha13 | 2018 | 17 M 0713259 9561037 | n.a. | natural regeneration |
| Pinus | *Heliocarpus americanus* | | ha14 | 1979 | 17 M 0713302 9561053 | n.a. | natural regeneration |
| Pinus | *Tabebuia chrysantha* | | tc01 | 2032 | 17 M 0713276 9561156 | G4 | Species subplot ^C^ |
| Pinus | *Tabebuia chrysantha* | | tc02 | 2033 | 17 M 0713274 9561155 | G4 | Species subplot ^C^ |
| Pinus | *Tabebuia chrysantha* | | tc03 | 1983 | 17 M 0713235 9561032 | n.a. | natural regeneration |
| Pinus | *Tabebuia chrysantha* | | tc04 | 2045 | 17 M 0713276 9561159 | G4 | Species subplot ^C^ |
| Pinus | *Tabebuia chrysantha* | | tc05 | 2025 | 17 M 0713237 9561100 | P3 | Species subplot ^C^ |
| Pinus | *Tabebuia chrysantha* | | tc06 | 2025 | 17 M 0713270 9561160 | G4 | Species subplot ^C^ |
| Pinus | *Tabebuia chrysantha* | | tc07 | 2041 | 17 M 0713276 9561160 | G4 | Species subplot ^C^ |
| Pinus | *Tabebuia chrysantha* | | tc08 | 2042 | 17 M 0713248 9561126 | G3 | Species subplot ^C^ |
| Pinus | *Tabebuia chrysantha* | | tc09 | 2003 | 17 M 0713276 9561095 | P2 | Species subplot ^C^ |
| Pinus | *Tabebuia chrysantha* | | tc10 | 2018 | 17 M 0713279 9561092 | P2 | Species subplot ^C^ |
| Pinus | *Tabebuia chrysantha* | | tc11 | 2042 | 17 M 0713251 9561124 | G3 | Species subplot ^C^ |
| Pinus | *Tabebuia chrysantha* | | tc12 | 2031 | 17 M 0713247 9561127 | G3 | Species subplot ^C^ |
| Pinus | *Tabebuia chrysantha* | | tc13 | 2020 | 17 M 0713244 9561104 | P3 | Species subplot ^C^ |
| Pinus | *Tabebuia chrysantha* | | tc14 | 2041 | 17 M 0713239 9561093 | P3 | Species subplot ^C^ |
| Pinus | *Tabebuia chrysantha* | | tc15 | 2014 | 17 M 0713241 9561101 | P3 | Species subplot ^C^ |
| Pinus | *Tabebuia chrysantha* | | tc16 | 1996 | 17 M 0713285 9561063 | P1 | Species subplot ^C^ |
| Shrub | *Heliocarpus americanus* | | ha01 | 2111 | 17 M 0712139 9560260 | 234 | Single species |
| Shrub | *Heliocarpus americanus* | | ha02 | 2108 | 17 M 0712135 9560250 | 234 | Single species |
| Shrub | *Heliocarpus americanus* | | ha03 | 2145 | 17 M 0712086 9560360 | 116 | Mixed species ^A^ |
| Shrub | *Heliocarpus americanus* | | ha04 | 2170 | 17 M 0712059 9560410 | 111 | Mixed species ^B^ |
| Shrub | *Heliocarpus americanus* | | ha05 | 2112 | 17 M 0712134 9560260 | 234 | Single species |
| Shrub | *Heliocarpus americanus* | | ha06 | 2171 | 17 M 0712057 9560410 | 111 | Mixed species ^B^ |
| Shrub | *Heliocarpus americanus* | | ha07 | 2172 | 17 M 0712056 9560410 | 111 | Mixed species ^B^ |
| Shrub | *Tabebuia chrysantha* | | tc01 | 2131 | 17 M 0712131 9560310 | 250 | Single species |
| Shrub | *Tabebuia chrysantha* | | tc02 | 2130 | 17 M 0712127 9560310 | 250 | Single species |
| Shrub | *Tabebuia chrysantha* | | tc03 | 2190 | 17 M 0712160 9560490 | 7 | Single species |
| Shrub | *Tabebuia chrysantha* | | tc04 | 2191 | 17 M 0712157 9560500 | 7 | Single species |
| Shrub | *Tabebuia chrysantha* | | tc05 | 2189 | 17 M 0712155 9560490 | 7 | Single species |
| Shrub | *Tabebuia chrysantha* | | tc06 | 2190 | 17 M 0712161 9560490 | 7 | Single species |
| Shrub | *Tabebuia chrysantha* | | tc07 | 2189 | 17 M 0712155 9560490 | 7 | Single species |
| Shrub | *Tabebuia chrysantha* | | tc08 | 2142 | 17 M 0712090 9560360 | 117 | Single species |
| Shrub | *Tabebuia chrysantha* | | tc09 | 2129 | 17 M 0712200 9560300 | 281 | Single species |
| Shrub | *Tabebuia chrysantha* | | tc10 | 2190 | 17 M 0712157 9560490 | 7 | Single species |
| Shrub | *Tabebuia chrysantha* | | tc11 | 2221 | 17 M 0712069 9560520 | 38 | Single species |
| Shrub | *Tabebuia chrysantha* | | tc12 | 2219 | 17 M 0712071 9560510 | 38 | Single species |
| Shrub | *Tabebuia chrysantha* | | tc13 | 2151 | 17 M 0712168 9560350 | 158 | Single species |
| Shrub | *Tabebuia chrysantha* | | tc14 | 2129 | 17 M 0712202 9560300 | 281 | Single species |
| Shrub | *Tabebuia chrysantha* | | tc15 | 2129 | 17 M 0712205 9560300 | 281 | Single species |
| Shrub | *Tabebuia chrysantha* | | tc16 | 2130 | 17 M 0712202 9560300 | 281 | Single species |
| Shrub | *Tabebuia chrysantha* | | tc17 | 2144 | 17 M 0712092 9560360 | 117 | Single species |
| Shrub | *Tabebuia chrysantha* | | tc18 | 2218 | 17 M 0712072 9560510 | 38 | Single species |
| Shrub | *Tabebuia chrysantha* | | tc20 | 2190 | 17 M 0712157 9560490 | 7 | Single species |
|  |  | |  |  |  |  |  |
| * | | Plot numbers and types refer to the original plantation design outlined in Aguirre (2007). Sample trees chosen outside this original plantation setting cannot be associated with pre-existing plots and therefore do not have a plot number (marked as n.a.) | | | | | |
| Mixed species ^A^ | | *C. montana, H. americanus, Alnus acuminata* | | | | | |
| Mixed species ^B^ | | *H. americanus, Juglans neotropica* | | | | | |
| Species subplot ^C^ | | Plots were subdivided into 4×4 m subplots of the following species:  *Alnus acuminata*, *Cedrela montana*, *Cinchona officinalis, Cupania cf americana*, *Heliocarpus americanus, Isertia laevis, Myrica pubescens, Piptocoma discolor* and *Tabebuia chrysantha* | | | | | |
