# Supplementary material for: Low Herbivory among Targeted Reforestation Sites in the Andean Highlands of Southern Ecuador
Source: PLoS One. 2016 Mar 10;11(3):e0151277. doi: 10.1371/journal.pone.0151277 (PMC4786223; doi:10.1371/journal.pone.0151277)
Supplement: S2 Table — The numerals before and after the slash denote the number of trees exhibiting fresh leaves, and the total number of foliage-bearing trees, respectively. The corresponding percentage value is given in parentheses. The first survey is not taken into account since both deciduous species (i.e. C. montana and T. chrysantha) typically develop new foliage during this time. (DOCX) [file pone.0151277.s002.docx]

|  |  | | **Survey** | | | | | | | | |
| --- | --- | --- | --- | --- | --- | --- | --- | --- | --- | --- | --- |
| **Habitat** | **Tree sp.** | **N** | **1** | **2** | | **3** | | **4** | | **5** | |
| **Forest** | *C. montana* | 10 | --- | 0/10 | (0.0%) | 0/9 | (0.0%) | 0/8 | (0.0%) | 0/7 | (0.0%) |
|  | *H. americanus* | 13 | --- | 0/13 | (0.0%) | 0/13 | (0.0%) | 0/13 | (0.0%) | 0/12 | (0.0%) |
|  | *T. chrysantha* | 20 | --- | **3/20** | **(15.0%)** | 0/20 | (0.0%) | 0/16 | (0.0%) | 0/14 | (0.0%) |
| **Pinus** | *C. montana* | 15 | --- | 0/15 | (0.0%) | 0/15 | (0.0%) | 0/14 | (0.0%) | **1/12** | **(8.3%)** |
|  | *H. americanus* | 13 | --- | **1/13** | **(7.7%)** | 0/13 | (0.0%) | 0/13 | (0.0%) | 0/13 | (0.0%) |
|  | *T. chrysantha* | 16 | --- | 0/16 | (0.0%) | 0/16 | (0.0%) | 0/14 | (0.0%) | 0/13 | (0.0%) |
| **Pasture** | *C. montana* | 18 | --- | 0/18 | (0.0%) | 0/18 | (0.0%) | 0/18 | (0.0%) | 0/14 | (0.0%) |
|  | *H. americanus* | 11 | --- | 0/11 | (0.0%) | 0/10 | (0.0%) | 0/10 | (0.0%) | **1/11** | **(9.1%)** |
|  | *T. chrysantha* | 18 | --- | **1/15** | **(6.7%)** | **3/15** | **(20.0%)** | **1/8** | **(12.5%)** | **9/15** | **(60.0%)** |
| **Shrub** | *H. americanus* | 7 | --- | 0/7 | (0.0%) | 0/7 | (0.0%) | 0/7 | (0.0%) | 0/7 | (0.0%) |
|  | *T. chrysantha* | 19 | --- | 0/14 | (0.0%) | **4/17** | **(23.5%)** | **1/17** | **(5.9%)** | **2/16** | **(12.5%)** |
